# Space- and time-agnostic imaging of subwavelength electromagnetic sources

**DOI:** 10.1038/s41598-026-53114-1

**Published:** 2026-05-21

**Authors:** Elias Le Boudec, David Martinez, Hamidreza Karami, Farhad Rachidi, Marcos Rubinstein, Felix Vega

**Affiliations:** 1 Electromagnetic Compatibility Laboratory, EPFL, 1015 Lausanne, Switzerland; 2 Directed Energy Research Center, Technology Innovation Institute, Abu Dhabi, United Arab Emirates; 3 School of Engineering and Management Vaud, HES-SO University of Applied Sciences and Arts Western Switzerland, Yverdon-les-Bains, Switzerland

**Keywords:** Engineering, Optics and photonics, Physics

## Abstract

The imaging of electromagnetic interference sources in devices is an interesting tool for electromagnetic compatibility pre-compliance testing, but it poses three significant challenges: i) the sources size is often subwavelength, meaning that the diffraction limit hinders their imaging, and the ii) spatial and iii) temporal features of the sources are uncontrolled. Indeed, in space, their spurious propagation occurs in devices with arbitrary shapes and materials; and in time, they occur either because of unintended radiation from digital or analogue signals, or because of random pulsed events such as electrostatic discharges. To overcome these issues, we i) propose to use the time reversal technique in conjunction with a resonant metalens for subwavelength imaging of these interference sources. We report the first single-shot subwavelength image of an electrostatic discharge, enabling to distinguish radiation from two PCB traces spaced 8 mm apart. ii) To achieve device (space) agnosticism, the imaging method does not require simulation of the device or its environment thanks to a combination of frequency-domain data obtained through a scanner and time-domain data radiated from the device. iii) To ensure flexibility in the source frequency band, we present a novel design for a metalens that operates in a desired frequency band within the gigahertz range, thanks to effective medium theory.

## Introduction

Before reaching the market, all electronic devices must undergo electromagnetic compatibility (EMC) compliance testing. These tests verify, among other factors, that the device does not emit unwanted electromagnetic radiation from unintentional antennas. Additionally, products must demonstrate robustness against electrostatic discharges, as outlined, for example, in the IEC 61000-4-2 standard^1^. When a product fails radiation tests, identifying the specific components that require hardening can be challenging. Imaging solutions for electromagnetic interference sources can help reduce the time to market by accelerating the diagnostic process. Solutions to image electromagnetic interference sources and help designers achieve device immunity are desirable^2^ and can decrease the time to market of such products.

This source interference imaging can be done by direct measurement. Near-field scans are a way to achieve high spatial resolution and good sensitivity^3–5^ and have already been applied to large-scale integrated circuits^6^ and interference in a shielded enclosure^7^ including phaseless data^8^. Near-field data have also been applied to electrostatic discharges^9–11^. Unfortunately, these methods are still incompatible with single-shot imaging, i.e., imaging of unrepeatable sources (e.g., electrostatic air-discharges^12^ or when damage occurs). Other methods might be necessary in this case, such as post-failure optical images^13^.

Alternative methods are available to mitigate this issue, e.g., relying on indirect measurements such as emission source microscopy^14^, computational imaging of far-field data^15^, compressive sensing^16^, inverse filtering^17^, and synthetic aperture interferometric passive radiometry imaging^18^. A prevalent imaging approach is the time-reversal method^19^. This method relies on the measurement of radiated fields on what is called a “time-reversal mirror”^20^. This mirror consists of one^21^ to several sensors. The measurements are then time-reversed and propagated back into the medium from the time-reversal mirror. Theoretical analysis supported by experimental results shows that the fields converge to the original source location, allowing the image to be produced through various methods^22–24^. The method has been combined with deep transfer learning to locate lightning^25^, applied to transmission line networks^26^, combined with a correlation criterion and ray-tracing for reflective environments^27^, and used to locate interference sources in a resonant cavity^28^.

Since machine learning methods have been successfully applied to image processing, they have also been employed for electromagnetic source localisation and imaging. Huang and Fan^29^ show how the histogram of oriented gradients can be combined with support vector machine to determine dominant source dipole moments. Shu et al.^30^ use an artificial neural network to correct Green’s free-space function with diffraction and reflection effects. Khodaveisi et al.^31^ show how several machine learning methods fare for partial discharge localisation in power transformer tanks. Finally, convolutional neural networks have been applied to the radiation from a metamaterial designed to enhance imaging resolution^32^.

Classical imaging methods are diffraction-limited: they are not capable of discerning two point-sources closer than half a wavelength. Studies of methods designed to achieve subwavelength imaging, also known as “super-resolution,” have involved time reversal^33^. A path of interest involves metamaterials designed to increase imaging resolution, such as the resonant metalens^34–36^. These metalenses have been shown to work also with a single receiving antenna^37^. The appeal of the time-reversal method compared with traditional lens imaging with a resonant metalens^38^ is the possibility to place the time-reversal mirror at arbitrary locations, offering greater flexibility in the experimental setup, including imaging of three-dimensional sources. Modifications have been proposed for multi-frequency operation^39^ and planar versions based on split-ring resonators^40^. Other resolution-enhancement devices include the spherical geodesic waveguide^41^.

In this work, we propose a novel approach that combines the time reversal method with experimental backpropagation and a resonant metalens to image currents from interference sources, including electrostatic discharges. To the best of the authors’ knowledge, this is the first reported single-shot subwavelength imaging method for electrostatic discharges. Key contributions of the proposed method is the use of backpropagation data acquired experimentally in the frequency domain, combined with time-domain, single-shot oscilloscope data radiated from the device under test exposed to an electrostatic discharge. Indeed, unlike in other time-reversal-based methods, we do not use any simulation data. We argue that in real-world electromagnetic pre-compliance tests, the simulation burden of the device combined with its electromagnetic environment is too heavy for the method to be practically viable. Furthermore, to contrast with existing work^35^, the acquisition of the backpropagation data relies on a two-dimensional scan, which presents shielding challenges solved by a careful scanner design. Finally, we propose a straightforward, printed-circuit-based resonant metalens design that can be tailored for arbitrary resonant frequency in the gigahertz range thanks to effective medium theory. This is needed to ensure compatibility with uncontrolled excitations, which might have arbitrary frequency bands, such as electrostatic discharges.

This paper is structured as follows: first, we present in Sect. Methods the methods used to obtain subwavelength images through time-reversal with experimental backpropagation and a custom resonant metalens; next, Sect. Results presents the validation results for imaging a synthetic source as well as an electrostatic discharge; Sect. Discussion discusses the results, and Sect. Conclusion concludes this report.

## Methods

### Theoretical framework

To clarify the physical principles underlying the proposed imaging approach, we first establish its electromagnetic formulation. The method relies on Green’s functions and the time-reversal invariance of Maxwell’s equations in linear, reciprocal media.

#### Forward problem and Green’s function representation

Let $\textbf{J}(\textbf{r}',\omega )$ denote the current distribution on the device under test. The resulting electric field measured at position $\textbf{r}$ and angular frequency $\omega$ can be expressed using the dyadic Green’s function $\textbf{G}(\textbf{r},\textbf{r}',\omega )$ as

1 $$\begin{aligned} \textbf{E}(\textbf{r},\omega ) = \int _V \textbf{G}(\textbf{r},\textbf{r}',\omega )\,\textbf{J}(\textbf{r}',\omega )\, \textrm{d}\textbf{r}'. \end{aligned}$$

In the proposed experimental setup, the positions $\textbf{r}_i$ correspond to the antennas of the time-reversal mirror. The measured signals $s_i(\omega )$ at these locations are therefore proportional to

2 $$\begin{aligned} s_i(\omega ) \propto \int _V \textbf{G}(\textbf{r}_i,\textbf{r}',\omega )\,\textbf{J}(\textbf{r}',\omega )\, \textrm{d}\textbf{r}'. \end{aligned}$$

This expression highlights that the measured data encode the convolution of the source distribution with the Green’s function of the environment, which includes the effects of the cavity, the device under test, and the metalens when present.

#### Time-reversal focusing

In the frequency domain, time reversal consists in phase conjugating the recorded signals and re-emitting them into the medium. This corresponds to using $s_i^*(\omega )$ as excitation signals. The resulting backpropagated field at a point $\textbf{r}$ is given by

3 $$\begin{aligned} \textbf{E}_{\textrm{TR}}(\textbf{r},\omega ) \propto \sum _{i=1}^{N} \textbf{G}(\textbf{r},\textbf{r}_i,\omega )\, s_i^*(\omega ). \end{aligned}$$

Substituting (2) into (3) yields

4 $$\begin{aligned} \textbf{E}_{\textrm{TR}}(\textbf{r},\omega ) \propto \int _V \left[ \sum _{i=1}^{N} \textbf{G}(\textbf{r},\textbf{r}_i,\omega )\,\textbf{G}^*(\textbf{r}',\textbf{r}_i,\omega ) \right] \textbf{J}(\textbf{r}',\omega )\, \textrm{d}\textbf{r}'. \end{aligned}$$

The term in brackets acts as a spatial focusing kernel. In a lossless and reciprocal medium, the Green’s function satisfies

5 $$\begin{aligned} \textbf{G}(\textbf{r},\textbf{r}',\omega ) = \textbf{G}^T(\textbf{r}',\textbf{r},\omega ), \end{aligned}$$

which implies that the kernel in (4) is maximized when $\textbf{r} = \textbf{r}'$. Therefore, the time-reversed field refocuses at the original source location, forming the basis of the imaging process.

#### Subwavelength information and evanescent spectrum

The current distribution $\textbf{J}(\textbf{r},\omega )$ can be decomposed into spatial Fourier components characterized by transverse wavenumbers $k_t$. Components with $k_t > k_0 = \omega / c_0$ correspond to evanescent waves, whose amplitude decays exponentially with distance *z* from the source:

6 $$\begin{aligned} E(k_t, z) \sim e^{-k_t z}. \end{aligned}$$

These evanescent components carry subwavelength spatial information but are not accessible in the far field due to their rapid decay. As a result, conventional imaging systems are limited by diffraction.

#### Resonant metalens

The resonant metalens enables the recovery of subwavelength information by coupling evanescent fields to a set of discrete resonant modes. Each mode is associated with a specific spatial distribution and resonance frequency. Through near-field interaction, the subwavelength features of the source excite these modes, which then radiate into the far field with distinct spectral signatures.

This mechanism can be interpreted as a mapping between high spatial frequencies (large $k_t$) and measurable temporal or spectral variations. The time-reversal process exploits this mapping: by re-injecting the phase-conjugated signals, the system selectively excites the same resonant modes, leading to constructive interference at the original subwavelength features of the source. More information can be found in Subsection A flexible gigahertz-range resonant metalens.

### Imaging experimental setup

Based on the theoretical framework described above, the proposed imaging method consists of two main stages: a test stage and a backpropagation stage.

In the test stage, the radiation emitted by the device under test is recorded by a set of antennas that form the time-reversal mirror. These measurements capture the convolution of the source distribution with the Green’s function of the environment.

In the backpropagation stage, the Green’s functions between the time-reversal mirror and the image plane are experimentally measured using a scanning probe. These measurements enable the reconstruction of the field that would be obtained if the time-reversed signals were re-emitted into the medium. The combination of these two datasets allows the computation of the imaging functional defined in (10), without requiring any numerical simulation of the environment. When a resonant metalens is introduced, subwavelength spatial information carried by evanescent waves is encoded into measurable spectral variations. The time-reversal process then exploits this encoding to achieve focusing beyond the diffraction limit.

All experiments are performed in a 1 m^3^ aluminium-walled resonant cavity. While this cavity provides shielding against ambient electromagnetic noise, it also introduces resonances which do not permit field propagation below the lower cutoff frequency^42^. Our goal is to localise a radiation source on a device under test placed inside the resonant cavity. The device under test, pictured in Fig. 4(a), consists of a custom printed circuit board with pairs of transmission lines of various lengths and separations. In this experiment, we use a length of 50 mm and a separation of 8 mm. The board thickness is 1.6 mm and it is made of FR4. The bottom of the printed circuit board has no ground plane. There are radio-frequency connectors to energise the transmission lines. The core of the connector is connected to the top trace, and the shield to the bottom trace. The goal of the experiment is to determine which of two adjacent traces is active.

Both traces are connected to the exterior of the resonant cavity through coaxial cables. To avoid cable radiation that could affect the imaging resolution, both coaxial cables are placed inside a metallic sheath.

The proposed method relies on an experimental backpropagation from the time-reversal mirror to the image plane, i.e., the plane where the source is expected to be. Eight off-the-shelf, printed-circuit-board-based spiral antennas with a bandwidth from 800 MHz to 8.5 GHz form the time-reversal mirror. They are placed at convenient locations surrounding the source. The higher the number of antennas in the time-reversal mirror, the better the resolution—up to the diffraction limit. Indeed, redundancy reduces the effect of noise. Here, we choose eight channels as a trade-off between achieving a good resolution and maintaining a simple acquisition.

**Fig. 1:**
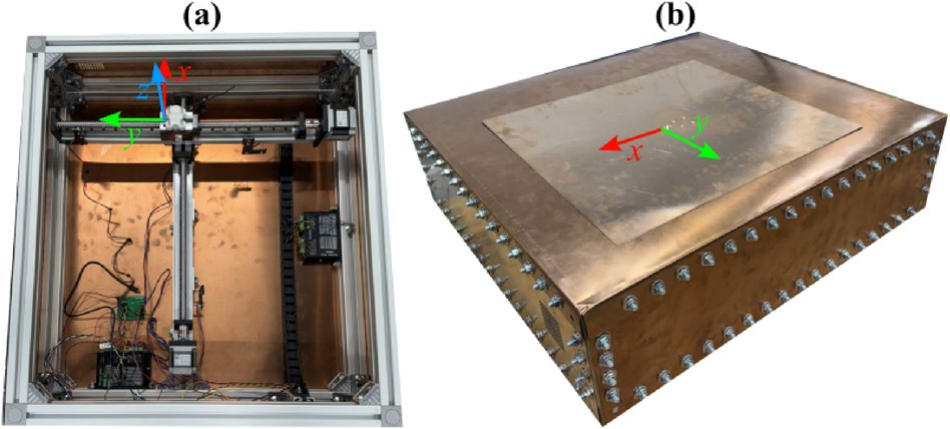
Scanner used to acquire backpropagation data. A 5 mm-long monopole antenna is mounted on a scanner housed in a copper-shielded box. (**a**): Stepper motors and controllers inside the scanner. (**b**): Shielded scanner.

To obtain the experimental data, we mount a 5 mm-long monopole antenna to a shielded *xy*-scanner, pictured in Fig. 1. The first resonance of this probe is well above the highest considered frequency of 3 GHz. The scanner consists of two ball-screw axes driven by stepper motors. The motors and their controllers are housed in a copper-shielded box. Both axes move the short monopole antenna along with a copper cover plate that seals the box aperture. This method is used to maintain the scanner geometry throughout its motion. Although the short monopole has a very low antenna factor when used below its resonance, it is appropriate due to its minimal physical footprint. Moreover, its low antenna factor also ensures that the distribution of electromagnetic fields inside the resonant cavity remains undisturbed by the antenna’s motion below 3 GHz, as verified by numerical simulations of the scanner. As such, it is assumed that the scanner voltage is proportional to the vertical electric field on the scanner’s surface below 3 GHz. The horizontal component of the field is negligible because of the scanner’s conductive shield. The range of motion is a 180 mm by 192.5 mm rectangle in steps of 25 $\upmu$m (although the mechanical tolerances exceed 25 $\upmu$m). This rectangle defines the image plane.

Across all experiments, we first establish an imaging baseline without the resonant metalens, allowing us to quantify the resolution improvement it provides.

The experiment runs in two stages: first, the test (or direct time) stage, and second, the backpropagation stage.

### Test stage

In this stage, the radiation from the device under test is recorded on the time-reversal mirror.

#### Synthetic excitation

Before applying the method to an electrostatic discharge, we test simpler excitations based on a Gaussian pulse. This allows us to evaluate the effects of signal frequency and bandwidth during postprocessing.

To this end, port $p=1,2$ of a vector network analyser (VNA) is connected to the corresponding trace of the device under test. Port 4 of the VNA is connected to an eight-channel bidirectional radio-frequency switch. We measure the scattering parameter from the device under test to channel $i=1,\dots ,8$ of the time-reversal mirror, i.e., $S_{4p}^i(f_n)$. The frequency $f_n$ is sampled uniformly from 100 kHz to 2999.6 MHz with 6,000 samples. The direct-time measurements signals are

7 $$\begin{aligned} s_p^i(f_n)=S_{4p}^i(f_n)g(f_n) \end{aligned}$$

where *g*(*f*) is the Fourier transform of a Gaussian pulse:

8 $$\begin{aligned} g(f)=\frac{1}{2\Delta f}\left[ e^{-2\left( \frac{f-f_0}{\Delta f/\pi }\right) ^2}+e^{-2\left( \frac{f+f_0}{\Delta f/\pi }\right) ^2}\right] \end{aligned}$$

**Fig. 2:**
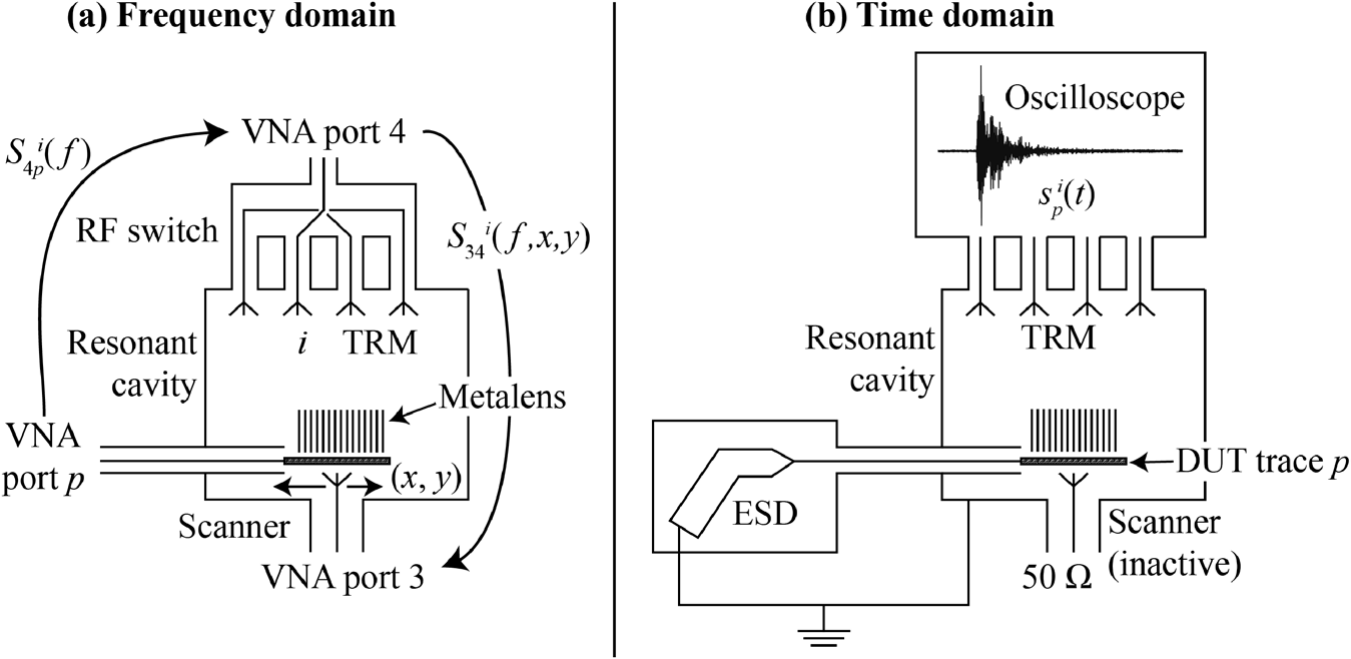
Schematics of the (**a**) frequency domain (synthetic excitation test stage and backpropagation stage) and (**b**) time-domain experiment setups. TRM: time-reversal mirror; VNA: vector network analyser; RF: radio-frequency; DUT: device under test.

$f_0$ is the centre frequency and $\Delta f$ the bandwidth. These parameters are adjusted manually depending on the experiment case. This test stage is described in Fig. 2(a).

#### Electrostatic discharge

We also test the radiation from the transient waveform caused by an electrostatic discharge. To generate this discharge, we use the calibration setup described in Annex B of IEC Standard 61000-4-2^1^. With an electrostatic voltage of 2 kV, we measure a rise time of 781 ps and a calculated voltage at the discharge target of 30.6 V.

We connect the discharge target to the active trace $p=1,2$ of the device under test in the resonant cavity thanks to a coaxial cable. A 16-channel oscilloscope with a sampling rate of 80 GS/s is used to synchronously capture the waveforms $s_p^i(t)$ radiated from the device under test to the time-reversal mirror channel $i=1,\dots ,8$. This test stage is described in Fig. 2(b). The peak voltage is sufficiently low to ensure that no dielectric breakdown occurs between neighbouring traces or between traces and the resonant metalens.

Finally, the time-domain waveforms $s_p^i(t)$ are transformed to the frequency domain (via an FFT) and multiplied by the Gaussian pulse *g*(*f*), obtaining $s_p^i(f)$. This multiplication is necessary to filter out the low and high frequencies and ensure control of the signal frequency band.

### Backpropagation stage

The goal of the backpropagation stage is to measure the field in the image plane (i.e., below the device under test) when the time-reversed measurements are emitted back from the time-reversal mirror.

To this end, the scanner is positioned at the coordinates (*x*, *y*) in a 15-by-15 rectangular grid of side 100 mm. Using the vector network analyser, we measure the scattering parameters $S_{34}^{i}(f_n,x,y)$, where port 3 is connected to the scanner and port 4 to the *i*th channel of the radio-frequency switch connected to the time-reversal mirror. This backpropagation stage is illustrated in Fig. 2(a).

**Fig. 3:**
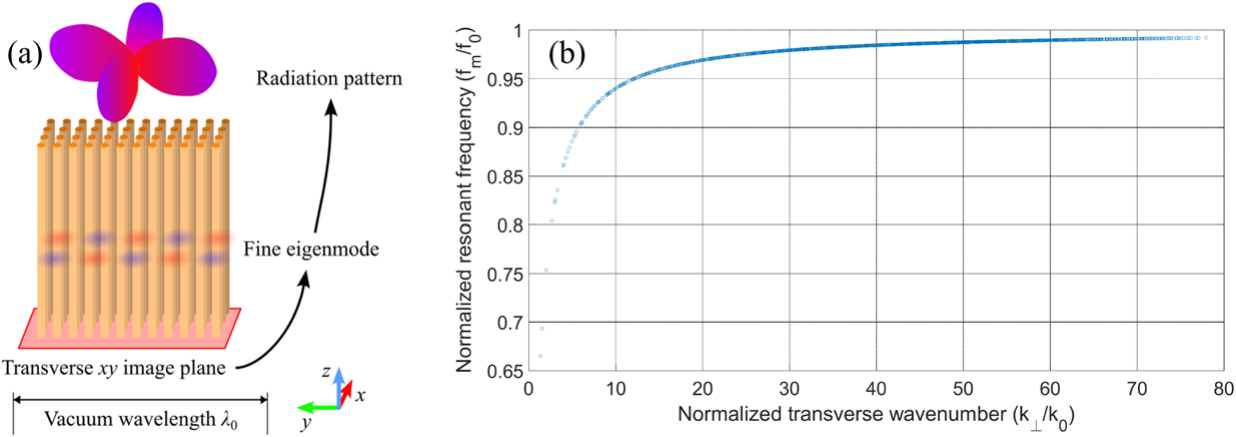
(**a**) Working principle of a resonant metalens. (**b**) Dispersion diagram of the resonant metalens.

Therefore, the vertical component of the time-reversed electric field in the image plane $r_p(f_n,x,y)$ is given by the sum over the time-reversal mirror channels

9 $$\begin{aligned} r_p(f_n,x,y)=\sum _{i=1}^8 S_{34}^{i}(f_n,x,y)[s_p^i(f_n)]^* \end{aligned}$$

where $\cdot ^*$ denotes complex conjugation.

Finally, to perform the imaging, we need a function of the position (*x*, *y*) alone. To this end, we sum over all frequencies $f_n$ and take the normalised quantity

10 $$\begin{aligned} r_p'(x,y)=\left[ \frac{\sum _{n}\left| r_p(f_n,x,y)\right| ^2}{\sum _{i,n}\left| S_{34}^i(f_n,x,y)g(f_n)\right| ^2}\right] ^{1/2} \end{aligned}$$

The denominator in Equation 10 acts as a normalisation (which is independent of the direct-time waveforms $s_p^i(f_n)$), similar to the “sensitivity compensation” in the supplementary information of Ref.^33^. It often provides clearer images by factoring out field enhancements caused by metal close to the scanner monopole antenna.

### A flexible gigahertz-range resonant metalens

The time-reversal method introduced above, as all classical imaging methods, is diffraction limited. In other words, two sources placed closer than $\lambda _0/2$ (where $\lambda _0$ is the working frequency) are expected to appear as a single one placed at the average of both source locations. For electrostatic discharges, where most of the energy is well below 1 GHz^43^, typical wavelengths are larger than $\lambda _\text {min}\approx 300\text { mm}$ and we expect a spatial resolution worse than $\lambda _\text {min}/2=150\text { mm}$. This resolution is of the same order of magnitude as the size of typical devices tested for electrostatic discharges.

To overcome this limit, we propose to use a metamaterial tailored for imaging resolution enhancement. One option is the resonant metalens, introduced by Lemoult et al.^34,36^. Its operating principle, illustrated in Fig. 3, is as follows. First, a current distribution with subwavelength details is generated in the image plane (*xy* plane below the metalens). Second, there exists a near-field coupling between the image plane and the fine eigenmodes inside the metalens. Third, these eigenmodes possess distinct radiation patterns at specific frequencies. Finally, these radiation patterns can be picked up in the metalens far-field and mapped back to the subwavelength image, thanks to the time-reversal method.

The resonant metalens consists of a square lattice of metallic rods of the same length. Its characteristics depend on the following parameters^35^:

- The operating bandwidth is defined by the resonant frequency
  11 $$\begin{aligned} f_0=c_0/(2\ell ) \end{aligned}$$
  where $c_0$ is the speed of light and $\ell$ is the length of the rods. In practice, the bandwidth of the resonant metalens lies between the first resonance (depending on the transverse size of the metalens) and a high cutoff frequency below $f_0$, depending on losses.
- The resolution is limited by the pitch between two adjacent rods.
- The resolution is limited by the losses inside the metalens. In practice, this limitation trumps the pitch resolution limit.

Therefore, the design of a metalens focuses on the minimisation of these losses.

In the megahertz range, a resonant metalens can be built by cutting copper rods to length and placing them between low-loss dielectric spacer plates. In the gigahertz range, the pitch is in the millimetre range and this method is no longer practical. Kaina et al.^44^ used copper plating on a 3D-printed support. Here, we introduce an alternative, inexpensive, mechanically robust, and simple manufacturing process. The rationale for this process is to be able to quickly iterate prototypes with resonant frequencies adapted for the imaged source. This is crucial for electrostatic discharges: these events have wideband and complex characteristics^43^ that are, moreover, uncontrolled. Hence, the choice of working frequency range is central to obtaining a sufficient transient measurement signal-to-noise ratio.

**Fig. 4:**
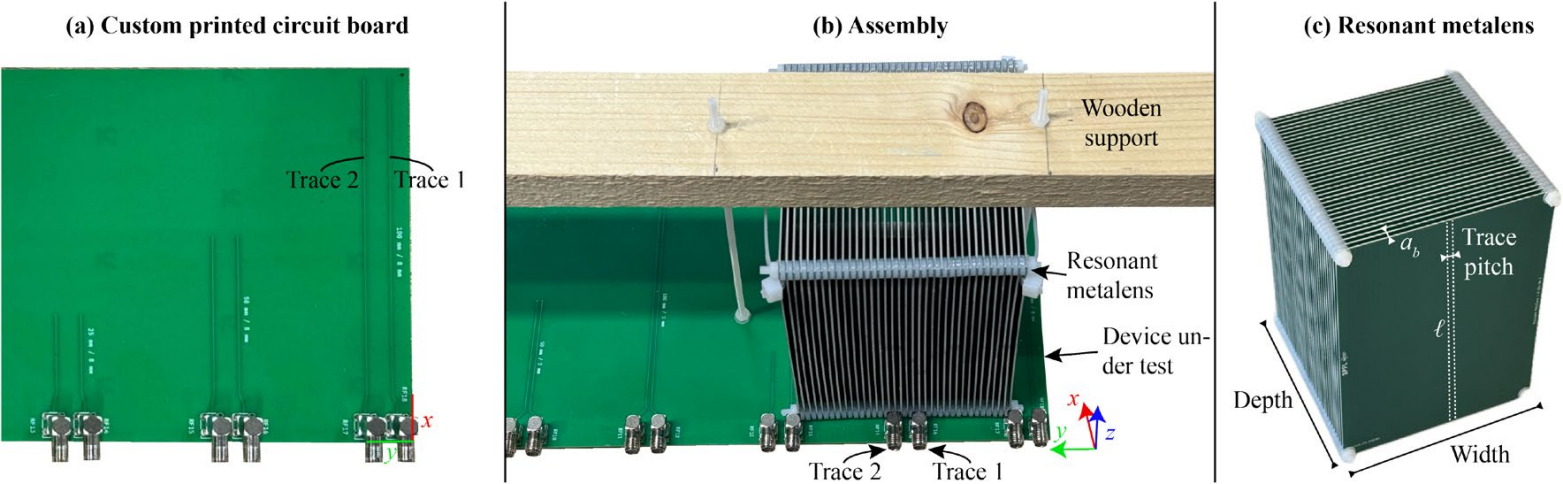
(**a**) Picture of the custom printed circuit board with the active traces. (**b**) Picture of the resonant metalens placed on top of the device under test. (**c**) Picture of the resonant metalens. The position of two traces is highlighted by two dashed white lines.

This process involves using low-loss PTFE printed circuit boards (PCBs) onto which copper parallel traces of a thickness around 0.2 mm are etched. It should be noted that this type of PCB-based metalens implementation is more cost-effective, simpler, and more robust compared to the previously proposed structure presented in^34^. The characteristics of the metalens are summarised in Fig. 3, and an image is shown in Fig. 4(c).

The used manufacturing method requires adjusting the speed of light used to determine the resonant frequency $f_0$ in Equation11. Indeed, the presence of the dielectric PTFE substrate slows down electromagnetic waves as the permittivity is $\varepsilon _r=2.94$ at the considered frequency. To calculate the effective speed of light, we can use a relative effective permittivity by volumetric averaging:

12 $$\begin{aligned} \varepsilon _{r,\text {eff}}=\varepsilon _r \frac{t_b}{a_b}+1 \cdot \frac{a_b-t_b}{a_b} \end{aligned}$$

where $t_b=0.76$ mm is the board thickness and $a_b$ the board pitch. The corrected effective resonant frequency is then

13 $$\begin{aligned} f_{0,\text {eff}}=\frac{c_0}{2\ell \sqrt{\varepsilon _{r,\text {eff}}}} \end{aligned}$$

The calculated values are reported in Table 1 and have been experimentally verified as the higher resonance cutoff frequency. It should be noted that the unit-cell dimensions of the proposed metalens remain subwavelength over the operating frequency range, thereby supporting the validity of Equation (12) up to a few gigahertz. Moreover, the actual metalens response is obtained experimentally from transfer-function measurements, rendering the imaging process fully data-driven. To increase the imaging resolution with the device under test, the resonant metalens is placed on top of the device, as pictured in Fig. 4(b). The distance between the traces and the bottom of the resonant metalens is around 1 mm. This permits a near-field coupling between the active traces and the bottom face of the resonant metalens. As the coupling distance increases, the evanescent spectrum is progressively attenuated, and the system tends toward diffraction-limited behavior. This limitation is fundamental and is not specific to the proposed implementation; rather, it is intrinsic to all near-field super-resolution techniques based on evanescent-wave recovery. This point should be considered when evaluating applications involving enclosed or shielded devices.

It should be noted that the implemented metalens in Fig. 4(c) exhibits an absolute bandwidth of approximately 327 MHz, corresponding to a fractional bandwidth of 39.5%, which places it in the ultra-wideband category. This operational window (665–992 MHz) overlaps with the relevant spectral content of the ESD transient, enabling the metalens to capture a meaningful fraction of the radiated pulse energy. Consequently, multiple resonant contributions can be exploited for image formation, and the experimental results confirm that the available bandwidth is sufficient to achieve reliable source localization. Moreover, the metalens cutoff frequency is approximately 665 MHz; therefore, for an ESD spectrum extending up to 1 GHz, roughly 66% of the nominal 0–1 GHz bandwidth lies below cutoff and is not captured by the metalens response. Nevertheless, the relatively high quality factor of the cavity supports strong, well-separated resonances within the passband, making the proposed metalens practical for localization. In addition, emphasizing higher-frequency components is advantageous for spatial resolution, since shorter wavelengths provide finer effective imaging detail.

Figure 3(b) presents the dispersion diagram of the resonant metalens, where the modal resonance frequencies are plotted in normalized form versus the normalized transverse wavenumber for the structure depicted in Fig. 4(c). Both quantities are normalized with respect to the fundamental Fabry–Pérot resonance frequency $f_0$ and its associated wavenumber $k_0$. The dispersion diagram was calculated using the method presented in Ref.^34^.

**Table 1:** Characteristics of the resonant metalens. The parameters are illustrated in Fig. 4(c).

Parameter	Value
Width	80 mm
Depth	79.1 mm
Trace length $\ell$	116.7 mm
Trace pitch	2.0 mm
Board pitch $a_b$	2.26 mm
$f_{0,\text {eff}}$	1.0 GHz

## Results

**Fig. 5:**
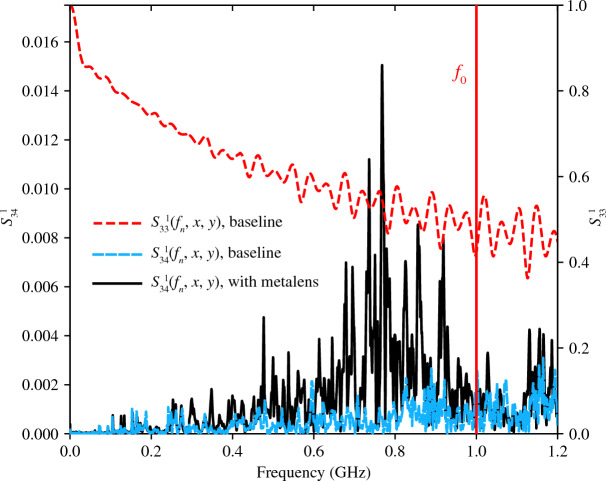
Example backpropagation scattering parameters between channel one of the time-reversal mirror and the scanner at $x=y=50~$mm with and without a resonant metalens. Dashed red: scanner return loss $S_{33}^1(f_n,x,y)$. Dashed blue (resp. solid black): transmission scattering parameter $S_{34}^1(f_n,x,y)$ without (resp. with) the resonant metalens. The vertical solid red line indicates the resonant frequency $f_0=1$ GHz.

**Fig. 6:**
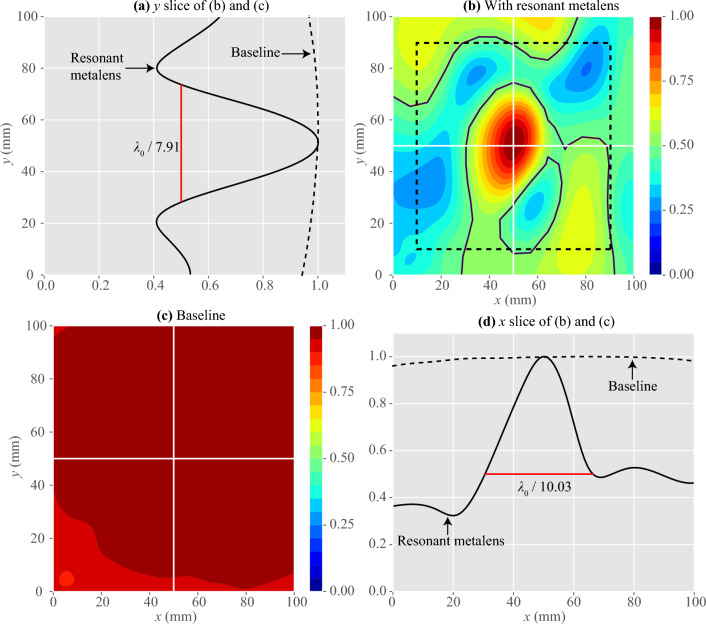
Max-normalised point source imaging of a synthetic excitation. (**b**) With a resonant metalens and (**c**) without (baseline). In (**b**), the half-max isoline is indicated by a black solid line, and the dashed black square marks the position of the resonant metalens. (**a**) *y* slice of the image at the original source location, $x=50$ mm. (**d**) *x* slice of the image at the original source location, $y=50$ mm. (a, d): solid line: with a resonant metalens; dashed line: without. In both cases, $f_0=835$ MHz, $\Delta f=130$ MHz.

**Fig. 7:**
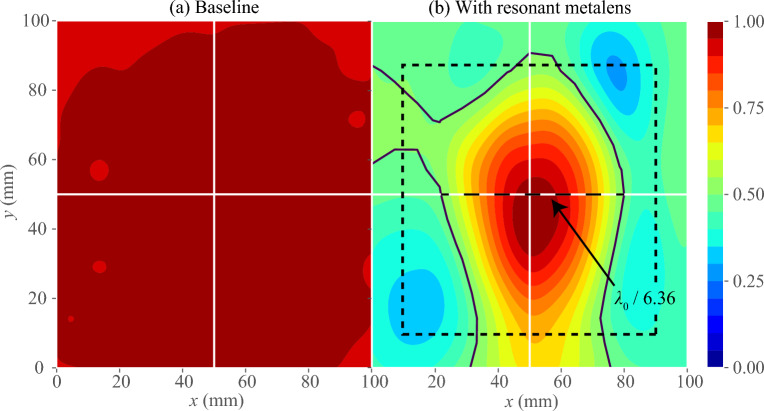
Point source imaging of an electrostatic discharge. (**a**): Empty chamber (baseline); (**b**): with a resonant metalens on top of the scanner. The half-max isoline is indicated by a black solid line, and the dashed black square marks the position of the resonant metalens. In both cases, $f_0=800$ MHz, $\Delta f=400$ MHz.

An example of measured scattering parameters is shown in Fig. 5, and a qualitative example of time-domain oscilloscope data is shown in the inset in Fig. 2(b). To verify the increase in resolution with the resonant metalens, Fig. 6 shows the image obtained without the device under test, when the scanner monopole antenna acts as a synthetic excitation source placed at $x_0=y_0=50\text { mm}$. The antenna is then supplied with an electrostatic discharge, and we obtain the image in Fig. 7. In both cases, we compare the image with and without the resonant metalens placed a few millimeters above the scanner monopole antenna.

**Fig. 8:**
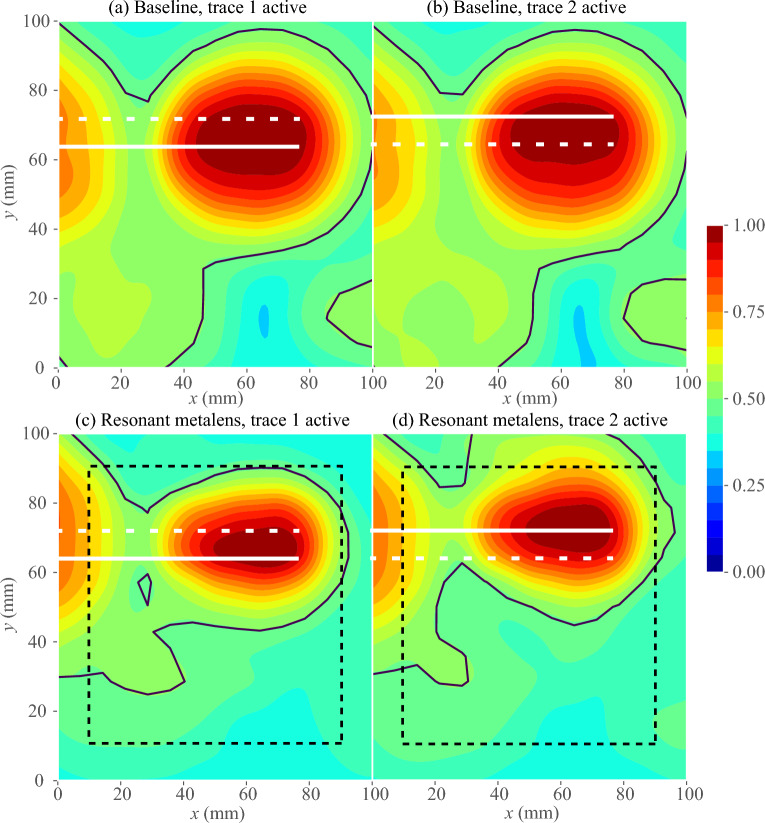
Max-normalised imaging results $r_p'(x,y)$ as in Equation 10 for a synthetic excitation in the device under test. (**a**, **b**): baseline, (**c**, **d**): with a resonant metalens. (a, c): trace 1 active. (b, d): trace 2 active. The location of the active trace is indicated by a solid white line, the other trace is the dashed white line. In all cases, $f_0=$ 1,100 MHz, $\Delta f=$ 1,800 MHz, and the half-max isoline is indicated by a black solid line, and the dashed black square marks the position of the resonant metalens.

**Fig. 9:**
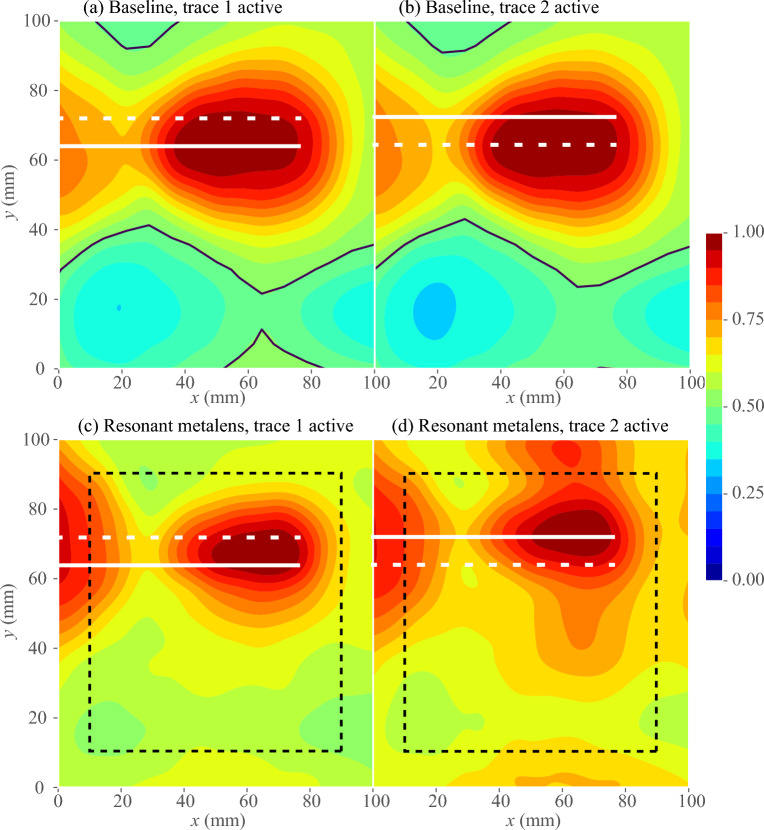
Max-normalised imaging results $r_p'(x,y)$ as in Equation 10 for an electrostatic discharge in the device under test. (**a**, **b**): baseline, (**c**, **d**): with a resonant metalens. (**a**, **c**): trace 1 active. (**b**, **d**): trace 2 active. The location of the active trace is indicated by a solid white line, the other trace is the dashed white line. In all cases, $f_0=$ 1,100 MHz, $\Delta f=$ 1,800 MHz, the half-max isoline is indicated by a black solid line, and the dashed black square marks the position of the resonant metalens.

**Fig. 10:**
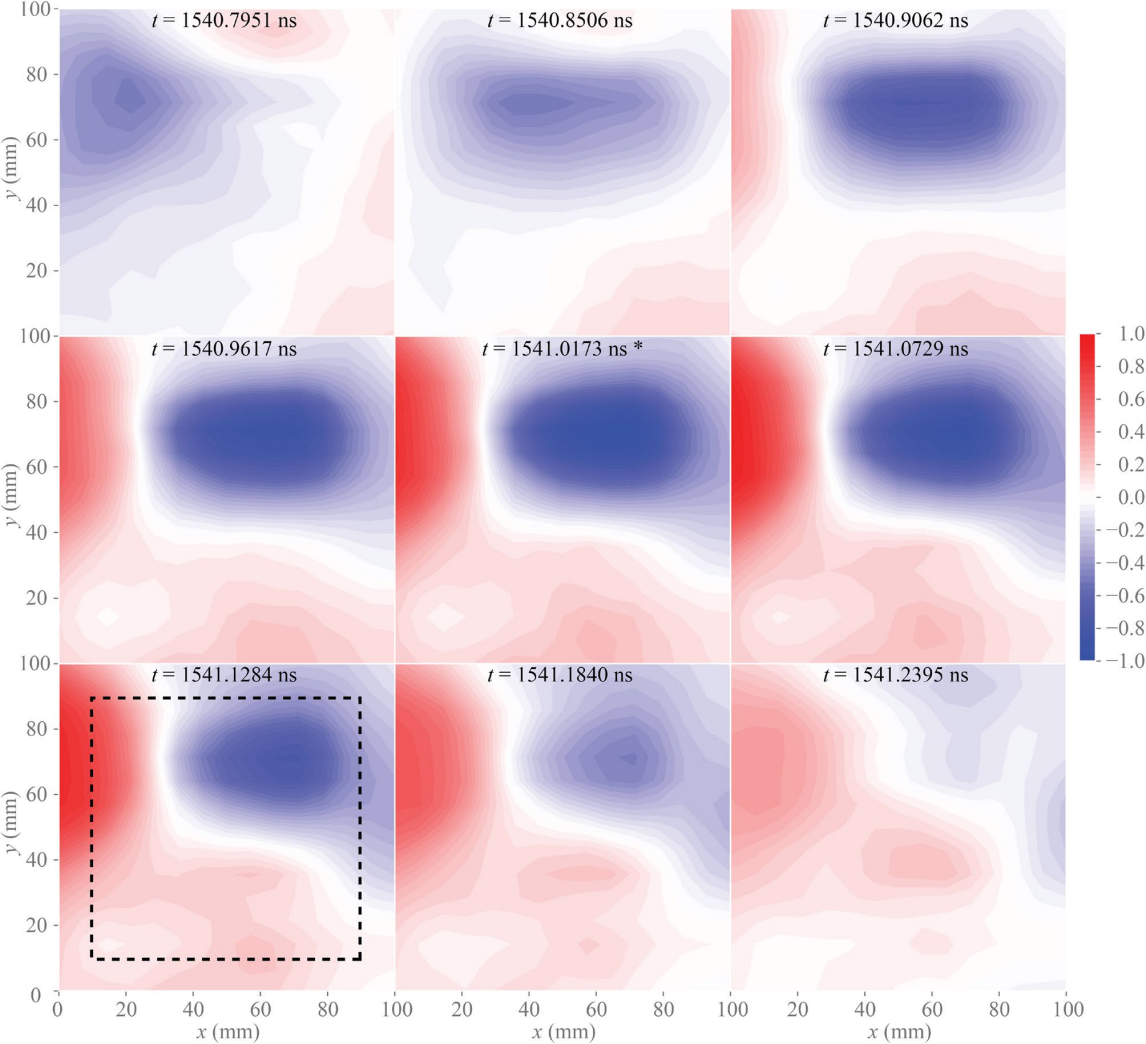
Time slices of the vertical electric field distribution $r_1(t,x,y)$ around the focusing time (i.e., the time of maximum amplitude, indicated by *) for the electrostatic discharge on trace 1 with a resonant metalens. The dashed black square marks the position of the resonant metalens.

Next, we place the device under test above the scanner monopole antenna and obtain the images in Fig. 8 for a synthetic excitation and Fig. 9 for an electrostatic discharge. Finally, Fig. 10 shows time slices of the vertical electric field obtained after an inverse Fourier transform of $r_1(t,x,y)$ in Equation9 around the focusing time (i.e., the time where the maximum amplitude is obtained).

## Discussion

Without the resonant metalens, and with the spatial window of 100 mm by 100 mm reported in the results, we do not expect to be able to localise a point source at $x_0=y_0=50\text { mm}$. Indeed, the expected focal spot size at the centre frequency $f_0$ and wavelength $\lambda _0=c_0/f$ is $\lambda _0/2=180\text { mm}$. Indeed, the image in Fig. 6(c) is roughly constant. However, when the resonant metalens is introduced, the field is maximised at the original source location (Fig. 6b), and we observe a full width at half maximum of $\sim \lambda _0/10$ in the *x* axis (Fig. 6d) and $\sim \lambda _0/8$ in the *y* axis (Fig. 6a). It should be noted that, since the proposed imaging framework is linear and time-invariant (see Sect. Theoretical framework), the reconstructed field for an arbitrary source distribution can be expressed as the convolution of the source with the system point-spread function. Consequently, the spatial resolution is fully characterized by the response to a single point source, and metrics such as the full width at half maximum provide a sufficient and rigorous measure of the system’s resolving capability. When the source is an electrostatic discharge, the same behaviour is observed in Fig. 7—i.e., the source becomes visible only with the resonant metalens. However, there is a deterioration of the obtained resolution: the full width at half maximum is $\sim \lambda _0/6$ in the *x* axis, and undetermined in the *y* axis. This is caused by a lower signal-to-noise ratio in the oscilloscope data, crosstalk between the electrostatic discharge simulator and the oscilloscope, and a lack of control of the excitation signal for the case of an electrostatic discharge. Moreover, for these time-domain experiments, the overall system is not strictly reciprocal (a requirement for time-reversal invariance in passive media^45^) because we used simultaneous acquisitions in the test stage, and sequential acquisitions using a switch in the backpropagation stage. As such, the switch imperfections (insertion loss and crosstalk) are not taken into account. Another source of loss of time-reversal invariance is the presence in the device under test of non-linear elements excited by the high-voltage electrostatic discharge event. However, this non-linearity is believed to have negligible effects on the results. Indeed, during the test stage, the radiated electric field from a device containing nonlinear elements is determined by the effective current densities. This radiation process remains linear and generally occurs in a lossless or low-loss medium. During the backpropagation stage, the weak radiation efficiency of the device under test means that non-linear elements have a minimal impact on the scattered field.

Next, the imaging of the device under test trace radiation with a synthetic excitation shows that the general location of the active trace can be determined in Fig. 8(a, b). However, as expected in the absence of the resonant metalens, we are not capable of determining which trace is active. When introducing the resonant metalens in Fig. 8(c, d), the active trace becomes visible. The resonances caused by the introduction of the metalens are visible in the transmission scattering parameters in Fig. 5. This resolution improvement holds also with the electrostatic discharge in Fig. 9, and, as for point source imaging, we see a decrease in resolution caused by the less accurate data acquisition. There is a mismatch between the image maximum and the lower trace in Figs. 8 and 9(c), likely attributed to setup imperfections (tilt in the device under test varying the scanner-to-device distance across the image plane, scanner zero-position calibration error) and a trace separation close to the system resolution limit (the *y*-axis full width at half maximum in Fig. 6(b) is $\sim 45$ mm, higher than the trace separation of 8 mm).

Note that when the device under test is introduced, the imaging resolution is better than what could be expected from the point source imaging experiments in Figs. 6 and 7 without the resonant metalens. In these cases, the full width at half maximum cannot be established since it lies outside the imaged region. However, by introducing the device under test, the vertical full width at half maximum without the resonant metalens (Fig. 8a, b) is approximately 65 mm, i.e., $\lambda /4.2$ at the centre frequency of 1,100 MHz. This increase in resolution compared to Figs. 6 and7 can be attributed, on the one hand, to the resolution enhancement caused by the device under test itself (specifically, the parallel traces act as a rudimentary resonant metalens), and on the other, to the increase in measured field level. Indeed, the scanner monopole antenna has a low antenna factor below 3 GHz. The near-field coupling with the metallic traces in the device under test provokes a resonance red-shift and significantly increases the field levels measured at the time-reversal mirror.

Finally, Fig. 10 shows the time-domain field distribution in the image plane, without normalisation. The clear increase in field amplitude is correlated with a spatial focusing at the trace location, which is expected from the time-reversal method. This figure shows the single-shot equivalent of the propagation plots in refs.^9,11^: it is the time-reversed image of the fields propagating on the device at a subwavelength scale resulting from an electrostatic discharge.

## Conclusion

We have introduced a method to image the radiating current sources based on time reversal and applied it to the imaging of the current in a device under test caused by an electrostatic discharge and a synthetic excitation. We used a resonant metalens to obtain images below the diffraction limit. The working frequency range of this printed-circuit-board-based metalens was designed according to effective medium theory. The possibility of adjusting the frequency range of the metalens is necessary for imaging complex, wideband, uncontrolled sources such as electrostatic discharges. To the best of our knowledge, these results provide the first single-shot subwavelength imaging of an electrostatic discharge. The proposed approach combines time- and frequency-domain measurements and avoids any prior modeling or simulation of the device under test or its electromagnetic environment by leveraging experimentally acquired two-dimensional scan data. The backpropagation operator is derived entirely from measurements, in contrast to many state-of-the-art methods that rely on numerically computed Green’s functions, ray tracing, or geometry-specific learned models.

While the proposed method is already approaching a working prototype, future tests should focus on investigating devices under test with greater complexity. In particular, three-dimensional scans might be of interest depending on the device under test geometry. In that case, the metamaterial would also need a non-planar focal surface, such as a cube with each face featuring a resonant metalens. The use of a split-ring-resonator-based, planar resonant metalens could also improve the practicality of the measurement. Improvements would also be likely with a higher-gain near-field probe. Additionally, an aligned picture of the device under test could be combined with the field image to have a better understanding of the locations that are responsible for interference. The current implementation has practical limitations, including the need for (i) a resonant cavity, (ii) preliminary spatial scanning to acquire backpropagation data, and (iii) specialized measurement equipment. These constraints may limit immediate applicability in standard EMC diagnostics. Future work will focus on improving deployability by reducing system complexity.

Finally, the presented method mixing frequency-domain data acquired from a scan and transient data acquired indirectly through few sensors in complex environments (e.g., with metamaterials, high resonances) could be applied to other problems, such as the coupling between lightning electromagnetic fields and substations, partial discharge localisation, ultra-wide-band radar, medical imaging, or time interfaces.

## Supplementary Information

Supplementary Information.

## Data Availability

The data supporting the findings are publicly available [46], together with the code used to obtain the results [47].
