# Supplementary material for: Space- and time-agnostic imaging of subwavelength electromagnetic sources
Source: Sci Rep. 2026 May 21;16:23118. doi: 10.1038/s41598-026-53114-1 (PMC13396469; doi:10.1038/s41598-026-53114-1)
Supplement: Supplementary file 1 — Supplementary Information. [file 41598_2026_53114_MOESM1_ESM.pdf]

Supplemental information in support of "Space- and Time-Agnostic Imaging of Subwavelength Electromagnetic Sources"

Elias Le Boudec[1,*], David Martinez[2], Hamidreza Karami[3], Farhad Rachidi[1], Marcos Rubinstein[3], and Felix Vega[2]

[1]Electromagnetic Compatibility Laboratory, EPFL, Lausanne, 1015, Switzerland
[2]Directed Energy Research Center, Technology Innovation Institute, Abu Dhabi, United Arab Emirates
[3]Institute for Information and Communication Technologies, University of Applied Sciences and Arts Western Switzerland, Yverdon-les-Bains, 1401, Switzerland
[*]elias.leboudec@alumni.epfl.ch

July 18, 2025

S1 List of equipment

The following equipment was used in the experiment:

- Vector network analyser (VNA): Rhode & Schwarz ZNB 20
- Radio-frequency switch: Analog Devices HMC321ALP4E
- Oscilloscope: Teledyne Lecroy, consisting of four LabMaster 10-20Zi-A modules controlled by a LabMaster MCM-Zi-A master control module
